# New insight and metrics to understand the ontogeny and succession of *Lactobacillus plantarum* subsp. *plantarum* and *Lactobacillus plantarum* subsp. *argentoratensis*

**DOI:** 10.1038/s41598-018-24541-6

**Published:** 2018-04-16

**Authors:** Yong Ju Jin, Yu Kyoung Park, Min Seok Cho, Eui Seok Lee, Dong Suk Park

**Affiliations:** 1 Department of Agricultural Biotechnology, National Institute of Agricultural Sciences, Rural Development Administration, Jeonju, 54874 Republic of Korea; 2 Department of Oral and Maxillofacial Surgery, Guro Hospital, Korea University, Seoul, 08308 Republic of Korea

## Abstract

*Lactobacillus plantarum* is one of the most extensively studied *Lactobacillus* species because of its presence in a variety of environmental niches, versatility, and metabolic capabilities, resulting in the use of this organism in many industrial applications. However, although extensive effort has been invested in screening this species from a variety of habitats, a reliable and accurate method for studying the succession and ontogeny of this organism in complex ecosystems is still required to confirm the activity of *L. plantarum* at the subspecies level. Therefore, in this study, novel subspecies-specific genes for the quantitative detection of two *L. plantarum* subspecies were identified by comparative genomic analysis. The specificity of primer sets for selected genes specific to each targeted microbe was confirmed in kimchi samples. Interestingly, in all the kimchi samples at 4 °C, the presence of *L. plantarum* subsp. *argentoratensis* was not observed. Hence, we found that low temperatures markedly affected the ontogeny of *L. plantarum* subsp. *argentoratensis* during kimchi fermentation. Subsequently, this touchstone method will offer new insight and metrics to understand the ontogeny and succession of *L. plantarum* subsp. *plantarum* and *L. plantarum* subsp. *argentoratensis* in various niches.

## Introduction

*Lactobacillus plantarum* is a versatile and adaptable species encountered in a variety of environmental niches, including dairy and meat products, many vegetables, fermented plants and the gastrointestinal tracts of humans and animals^1^. *L. plantarum* is extensively used in the production of fermented foods, such as yogurt, cheese, kimchi, sauerkraut, sourdough, and pickles, and feed materials because it is considered a safe probiotic. In addition, *L. plantarum*, among many strains of lactic acid bacteria (LAB), is emerging as a strain for the improvement of skin health, such as treatment of itchy skin and for skin moisturization as well as intestinal health^2,3^.

Hence, there have been a relatively greater number of studies on the genomic diversity of *L. plantarum* strains than those of other LAB^1^. The association of the consumption of this *Lactobacillus* with greater longevity and improved health is the foundation for the development of this bacterium as a probiotic, for which the global market has greatly expanded over the last ten or more years^4^. The decade that followed the determination of the first genome sequence of a food-associated species, *L. plantarum*, saw the study of lactobacilli with a wide range of functional genomic approaches to identify the genes and gene products that regulate the distinctive phenotypes and health associations of these bacteria^5,6^.

However, prokaryotic systematics is currently reliant on labour- and time-intensive polyphasic taxonomic approaches, including DNA-DNA hybridization, analysis of variations in 16 S rRNA gene sequences and phenotypic characterization. Distinguishing between closely related species or subspecies using these techniques is difficult, often resulting in the misclassification and misidentification of bacterial strains^7,8^.

Moreover, the molecular and culture-dependent methods available for monitoring *L. plantarum* are also insufficient because these methods detect other species in the *L. plantarum* group as well, including *L. pentosus* and *L. fermentum*^4^. Consequently, there remains a lack of reproducibility of data associated with the change in density of specific LAB of interest upon various environmental changes at the species or subspecies level. Thus, due to the limitations of these prior studies, there is increasing demand for improvement of the current approaches to studying prokaryotic systems^9^.

Over the past two decades, with the development of microbial genome sequencing technology achieved via the hard work of many scientists, large amounts of genomic information have been generated and have provided new perspectives for microbiological classification and diagnosis, which has led to much progress in genome-based classification. However, many challenges remain in the identification of significant molecular markers for the ecological investigation of new probiotics or technologically attractive strains and for the evaluation of the physiological states of these strains to improve functionality during industrial processes^10^. For example, the beneficial effects of several LAB strains on host immune metabolic syndrome is not yet fully understood due to the absence of a comprehensive mechanistic explanation^11–14^.

*Lactobacillus* has played a unique role in the history of human culture and science. In particular, *L. plantarum* has been known to be a dominant LAB in the late stage of kimchi fermentation and is the most dominant microorganism at 20–30 °C. Kimchi is a traditional fermented Korean delicacy that is made from vegetables, including cabbage, and a range of spices and seasonings, and it is recognized as a health food worldwide. There are more than 200 diverse types of kimchi^15^.

Studies in many areas of the food industry have been focused on important LAB, such as *L. plantarum*, which is known to play a significant role in food fermentation, science, and industry in food microbiology^16^.

Consequently, in this study, multiple LAB genomes publicly available at the NCBI bacterial genomes ftp website (ftp://ftp.ncbi.nlm.nih.gov/genomes/genbank/bacteria/) were compared to identify a set of unique genes in the *L. plantarum* subspecies. Among the identified genes, we selected a cell surface protein gene to develop subspecies-specific primer sets for the identification and quantification of the two *L. plantarum* subspecies. The results of this study will provide insight into some long-held inaccurate beliefs regarding *L. plantarum* in various environments.

## Results

### Specificity tests of the selected genes and designed oligonucleotide primers

The selected genes and oligonucleotide primers (Table 1) for two *L. plantarum* strains, i.e., *L. plantarum* subsp. *plantarum* and *L. plantarum* subsp. *argentoratensis*, were evaluated and confirmed via two-step in-depth testing using the BLAST search engine (https://blast.ncbi.nlm.nih.gov/Blast.cgi) and the Lasergene PrimerSelect program (version 7.2.1; DNASTAR Inc., USA).

**Table 1:** Primer sequences, their targets, and the annealing temperatures used in *Lactobacillus plantarum* subsp. *plantarum* and *L. plantarum* subsp. *argentoratensis* PCR screens.

Primer	Oligonucleotide sequence (5′-3′)	Annealing (°C)	Amplicon (Region)	Target gene (GenBank accession no.)	Reference
T1PL186F	ACC CCC GTT CCG TCA GA	65	186 bp (8145~8330)	LPXTG-motif cell wall anchor domain protein (EFK28973.1)	This study
T1PL186R	ATC ACC GCT TCC CCG CTC ATT				
LPA187F	GCA TCC CGA CGC TAC TAC ACA	65	187 bp (10860~11046)	BspA family leucine-rich repeat surface protein (WP_054397841.1)	This study
LPA187R	GAT TTT ATT TGC GTC CCA CTC C				

In *L. plantarum* subsp. *plantarum*, the BLASTn searches presented no substantial match to known reference sequences from other *Lactobacillus* species. The BLASTx results using the predicted protein sequence revealed that the most similar protein to our cell surface protein was a *Halomonas campaniensis* protein [identity = 31%, score = 35.4 bits (80), and expect = 2.7], second only to the *L. plantarum* protein.

In *L. plantarum* subsp. *argentoratensis*, the BLASTn searches returned no robust match to other identified *Lactobacillus* reference sequences. The BLASTx results using the predicted protein sequence showed that there was no protein similar to our cell surface protein, with the exception of proteins from some *L. plantarum* strains.

**Figure 1:**
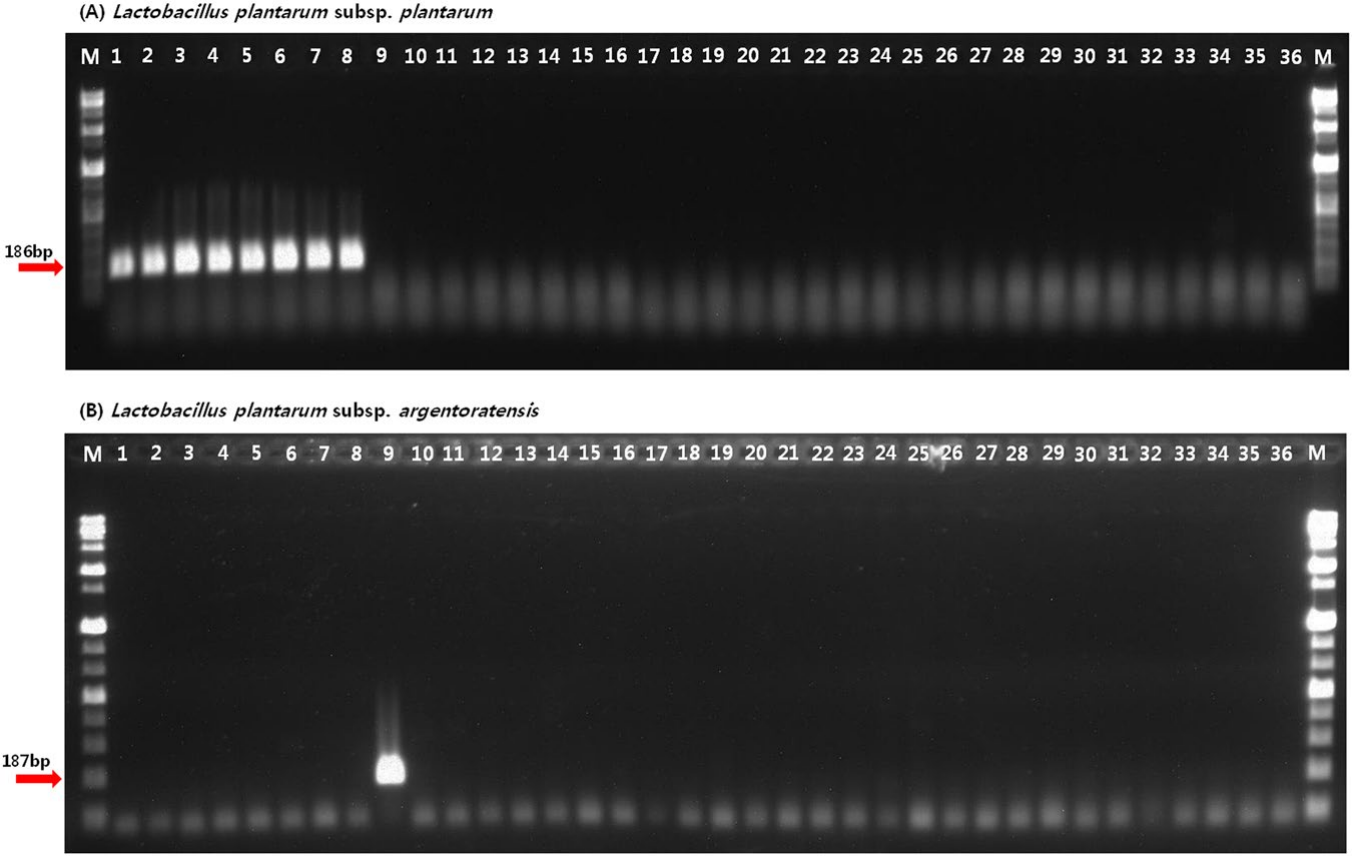
Specific PCR amplification of *Lactobacillus plantarum* subsp. *plantarum and L. plantarum* subsp. *argentoratensis* with the T1PL186F/R and LPA187F/R primer set. Lane M shows the size marker (1 kb Plus DNA ladder; Gibco BRL); lanes 1 to 8 contain *L. plantarum* subsp. *plantarum* samples; lane 9 contains a *L. plantarum* subsp. *argentoratensis* sample; lanes 10 to 36 contain samples of strains from other *Lactobacillus* species along with samples of strains from species of *Leuconostoc, Pediococcus, Streptococcus* and *Weissella*, as listed in Table 2.

In addition, DNA samples from LAB strains covering the type strains of each targeted subspecies (Table 2) were used to validate the specificity of each subspecies-targeting oligonucleotide primer set via a conventional PCR assay. Consequently, our *in silico* specificity tests confirmed that amplified products were observed only from the genomic DNA from *L. plantarum* subsp. *plantarum* and *L. plantarum* subsp. *argentoratensis* (Fig. 1).

**Table 2:** Bacterial strains used in this study.

Scientific Name	Source^a^	Biological origin	This study^b (T1PL186/LPA187)^
*Lactobacillus plantarum*	LMG 6907^T^	Pickled cabbage	+*/−*
*Lactobacillus plantarum*	LMG 9206	Dental caries	*+/*−
*Lactobacillus plantarum*	LMG 9208	Sauerkraut	*+/*−
*Lactobacillus plantarum*	LMG 12167	Homemade soft cheese	*+/*−
*Lactobacillus plantarum*	LMG 18024	Buffalo, Milk	*+/*−
*Lactobacillus plantarum*	LMG 18035	Fermented food from cassava	*+/*−
*Lactobacillus plantarum*	LMG 23521	Meat	*+/*−
*Lactobacillus plantarum*	LMG 25882	Dairy product	*+/*−
*Lactobacillus plantarum* subsp. *argentoratensis*	LMG 9205 ^T^	Fermented corn product(Ogi)	*−/+*
*Lactobacillus pentosus*	LMG 10775^T^	NK	*−/−*
*Lactobacillus paraplantarum*	KACC 12373^T^	Beer	*−/−*
*Lactobacillus acidophilus*	LMG 9433 ^T^	Human	*−/−*
*Lactobacillus amylolyticus*	LMG 18796^T^	Acidified beer wort	*−/−*
*Lactobacillus brevis*	LMG 6906^T^	Human	*−/−*
*Lactobacillus buchneri*	LMG 6892^T^	Tomato pulp	*−/−*
*Lactobacillus casei*	LMG 6904^T^	Cheese	*−/−*
*Lactobacillus crispatus*	LMG 9479^T^	Eye	*−/−*
*Lactobacillus delbrueckii* subsp. *bulgaricus*	LMG 6901^T^	Bulgarian yoghurt	*−/−*
*Lactobacillus fermentum*	LMG 6902^T^	Fermented beets	*−/−*
*Lactobacillus gasseri*	LMG 9203^T^	Human	*−/−*
*Lactobacillus hayakitensis*	LMG 24490^T^	Sealthy thoroughbred, faeces	*−/−*
*Lactobacillus helveticus*	KACC 12418^T^	Swiss Emmental cheese	*−/−*
*Lactobacillus sakei*	KACC 12414^T^	Starter of sake(Moto)	*−/−*
*Lactobacillus salivarius*	LMG 9477^T^	Saliva	*−/−*
*Lactococcus lactis*	KACC 13877^T^	NK	*−/−*
*Lactococcus lacits* subsp. *cremoris*	KACC 13438^T^	Cheese starter culture	*−/−*
*Leuconostoc citreum*	KACC 11860^T^	Honeydew of rye ear	*−/−*
*Leuconostoc fallax*	KACC 12303^T^	Sauerkraut	*−/−*
*Leuconostoc mesenteroides* subsp. *mesenteroides*	KACC 12312^T^	Fermenting olives	*−/−*
*Leuconostoc pseudomesenterodies*	KACC 12304^T^	Cane juice	*−/−*
*Pediococcus pentosaceus*	KACC 12311^T^	Dried American beer yeast	*−/−*
*Streptococcus gordonii*	KACC 13829^T^	Patient suffering from subacute bacterial endocarditis	*−/−*
*Weissella koreensis*	KACC 11853^T^	Kimchi	*−/−*
*Weissella minor*	KACC 13437^T^	Milking machine slime	*−/−*
*Weissella halotolerans*	KACC 11843^T^	Sausage	*−/−*
*Weissella thailandensis*	KACC 11849^T^	Fermented fish	*−/−*

^a^KACC, Korean Agricultural Culture Collection, Republic of Korea; LMG, The Belgian Co-ordinated Collections of Microorganisms (BCCM^TM^), Belgium.

^b^+, detected; −, not detected.

^T^Type of strain.

N.D., not determined.

These results demonstrate that the subspecies-specific primer pairs designed in this study are unique to each targeted *L. plantarum* subspecies and are adequate for the identification and quantification of these subspecies in various environments.

### SYBR Green real-time PCR: standard curves, detection and quantification limits

**Figure 2:**
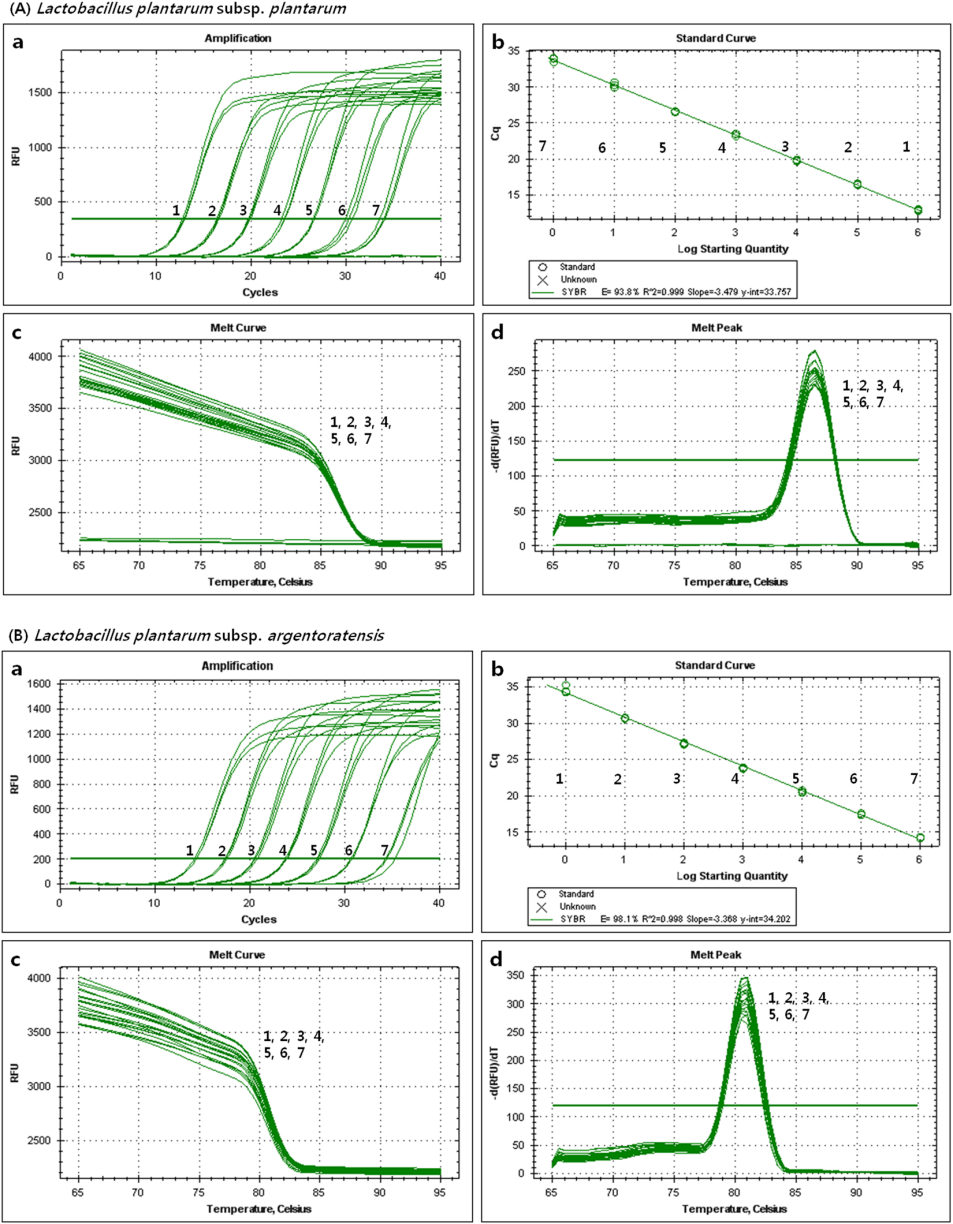
Specificity, melting peak and standard curve of the T1PL186F/R and LPA187F/R primer set by SYBR Green qPCR. (**A**) *Lactobacillus plantarum* subsp. *plantarum*. (a) Fluorescence intensity as a function of the template concentration. For each assay, a series of 10-fold dilutions of cloned DNA (ranging from 1.43 × 10^3^ to 1.43 × 10^9^ copies/µl) were used as template (1–7, sample dilutions). (b) Standard curve derived from the amplification plot. (c) Melting curve analysis (1–7, sample dilutions). (d) Melting peak analysis (1–7, sample dilutions). The derivatives of the relative fluorescence units of the amplified products [-d(RFU)/dT] were plotted as a function of temperature (amplified product, 86.5 °C). The high peak indicates the amplified product, and the low peak is the no-template control. (**B**) *Lactobacillus plantarum* subsp. *argentoratensis*. (a) Fluorescence intensity as a function of the template concentration. For each assay, a series of 10-fold dilutions of cloned DNA (ranging from 1.42 × 10^3^ to 1.42 × 10^9^ copies/µl) were used as template (1–7, sample dilutions). (b) Standard curve derived from the amplification plot. (c) Melting curve analysis (1–7, sample dilutions). (d) Melting peak analysis (1–7, sample dilutions). The derivatives of the relative fluorescence units of the amplified products [−d(RFU)/dT] were plotted as a function of temperature (amplified product, 81.5 °C). The intense peak indicates the amplified product.

SYBR Green real-time PCR was conducted to generate a standard curve with the type strain of each targeted subspecies (Tables 3 and 4), namely, *L. plantarum* subsp. *Plantarum* and *L. plantarum* subsp. *argentoratensis*, by plotting the mean threshold cycle (Ct) (n = 3) against the logarithmic concentrations of genomic DNA (*L. plantarum* subsp. *plantarum*, from 5 to 5 × 10^−5^ ng/µl; *L. plantarum* subsp. *argentoratensis*, from 5 to 5 × 10^−5^ ng/µl), cloned DNA (*L. plantarum* subsp. *plantarum*, from 1.43 × 10^9^ to 1.43 × 10^3^ copies/µl; *L. plantarum* subsp. *argentoratensis*, from 1.42 × 10^9^ to 1.42 × 10^3^ copies/µl) and cell suspension (*L. plantarum* subsp. *plantarum*, from 6.4 × 10^8^ to 6.4 × 10^3^ CFU/ml; *L. plantarum* subsp. *argentoratensis*, from 5.1 × 10^8^ to 5.1 × 10^5^ CFU/ml). The limit of quantitation (LOQ) assay presented an adequate linear response and an excellent correlation coefficient (*L. plantarum* subsp. *plantarum*, R^2^ = 0.999; *L. plantarum* subsp. *argentoratensis*, R^2^ = 0.998). A standard curve analysis on the linear portion of the slope yielded coefficients of −3.479 and −3.368, corresponding to PCR efficiencies of 93.8 and 98.1%, respectively, and y-intercept values of 33.757 and 34.202, respectively (Fig. 2). Melting analysis (curve, temperature, and peaks) of SYBR Green real-time PCR products from the above *Lactobacillus* species yielded reproducible melting temperatures (*L. plantarum* subsp. *plantarum*, 86.5 °C; *L. plantarum* subsp. *argentoratensis*, 81.5 °C) and specific peaks (Fig. 2). Standard curves of the input genomic DNA concentration (*L. plantarum* subsp. *plantarum*, R^2^ = 0.998, slope = −3.637; *L. plantarum* subsp. *argentoratensis*, R^2^ = 0.999, slope = −3.541) and the cell density (*L. plantarum* subsp. *plantarum*, R^2^ = 0.993, slope = −3.721; *L. plantarum* subsp. *argentoratensis*, R^2^ = 0.998, slope = −3.510) of each *L. plantarum* subspecies presented linear correlations with the corresponding cycle threshold (Ct) values. The detection limits of the SYBR Green real-time PCR assay for the input genomic DNA concentration was 50 fg/μl of reaction mixture, and the detection limits for the bacterial cell density were 6.4 × 10^3^ CFU/ml for *L. plantarum* subsp. *plantarum* and 5.1 × 10^5^ CFU/ml in *L. plantarum* subsp. *argentoratensis*.

**Table 3:** Mean cycle threshold (CT) end-point fluorescence of 10-fold serial dilutions of *L. plantarum* subsp. *plantarum* LMG 6907 genomic DNA, cloned DNA, and cell suspension as determined by the SYBR Green real-time polymerase chain reaction assay.

Genomic DNA		Cloned DNA		Cell suspension	
weight/${\boldsymbol{\mu }}{\boldsymbol{\ell }}$ reaction	C_*t*_ ± SD^a^ (n = 3)	Plasmid copies/${\boldsymbol{\mu }}{\boldsymbol{\ell }}$	C_*t*_ ± SD (n = 3)	CFU/ml reaction mix	C_*t*_ ± SD (n = 3)
5 ng	16.01 ± 0.12	1.43 × 10^9^	12.88 ± 0.12	6.4 × 10^8^	16.55 ± 0.06
500 pg	20.00 ± 0.10	1.43 × 10^8^	16.46 ± 0.10	6.4 × 10^7^	20.60 ± 0.05
50 pg	23.77 ± 0.24	1.43 × 10^7^	19.78 ± 0.12	6.4 × 10^6^	24.15 ± 0.07
5 pg	27.31 ± 0.18	1.43 × 10^6^	23.39 ± 0.11	6.4 × 10^5^	27.81 ± 0.21
500 fg	30.78 ± 0.33	1.43 × 10^5^	26.59 ± 0.05	6.4 × 10^4^	31.11 ± 0.28
50 fg	34.30 ± 0.30	1.43 × 10^4^	30.29 ± 0.35	6.4 × 10^3^	35.56 ± 1.50
5 fg	N.D.^b^	1.43 × 10^3^	33.85 ± 0.22	6.4 × 10^2^	N.D.

^a^SD, Three reactions standard deviation.

^b^N.D., Not detected.

**Table 4:** Mean cycle threshold (CT) end-point fluorescence of 10-fold serial dilutions of *L. plantarum* subsp. *argentoratensis* LMG 9205 genomic DNA, cloned DNA, and cell suspension as determined by the SYBR Green real-time polymerase chain reaction assay.

Genomic DNA		Cloned DNA		Cell suspension	
weight/${\boldsymbol{\mu }}{\boldsymbol{\ell }}$ reaction	C_*t*_ ± SD^a^ (n = 3)	Plasmid copies/${\boldsymbol{\mu }}{\boldsymbol{\ell }}$	C_*t*_ ± SD (n = 3)	CFU/ml reaction mix	C_*t*_ ± SD (n = 3)
5 ng	15.54 ± 0.20	1.42 × 10^9^	14.28 ± 0.06	5.1 × 10^8^	21.18 ± 0.23
500 pg	18.82 ± 0.09	1.42 × 10^8^	17.45 ± 0.12	5.1 × 10^7^	24.89 ± 0.08
50 pg	22.32 ± 0.12	1.42 × 10^7^	20.57 ± 0.18	5.1 × 10^6^	28.42 ± 0.11
5 pg	26.24 ± 0.02	1.42 × 10^6^	23.82 ± 0.09	5.1 × 10^5^	31.70 ± 0.11
500 fg	29.63 ± 0.17	1.42 × 10^5^	27.19 ± 0.12	5.1 × 10^4^	N.D.
50 fg	33.06 ± 0.28	1.42 × 10^4^	30.72 ± 0.09	5.1 × 10^3^	N.D.
5 fg	N.D.^b^	1.42 × 10^3^	34.66 ± 0.62	5.1 × 10^2^	N.D.

^a^SD, Three reactions standard deviation.

^b^N.D., Not detected.

### Quantitative detection of *Lactobacillus plantarum* subsp. *plantarum* and *Lactobacillus plantarum* subsp. *argentoratensis* in kimchi samples using SYBR Green real-time PCR

**Figure 3:**
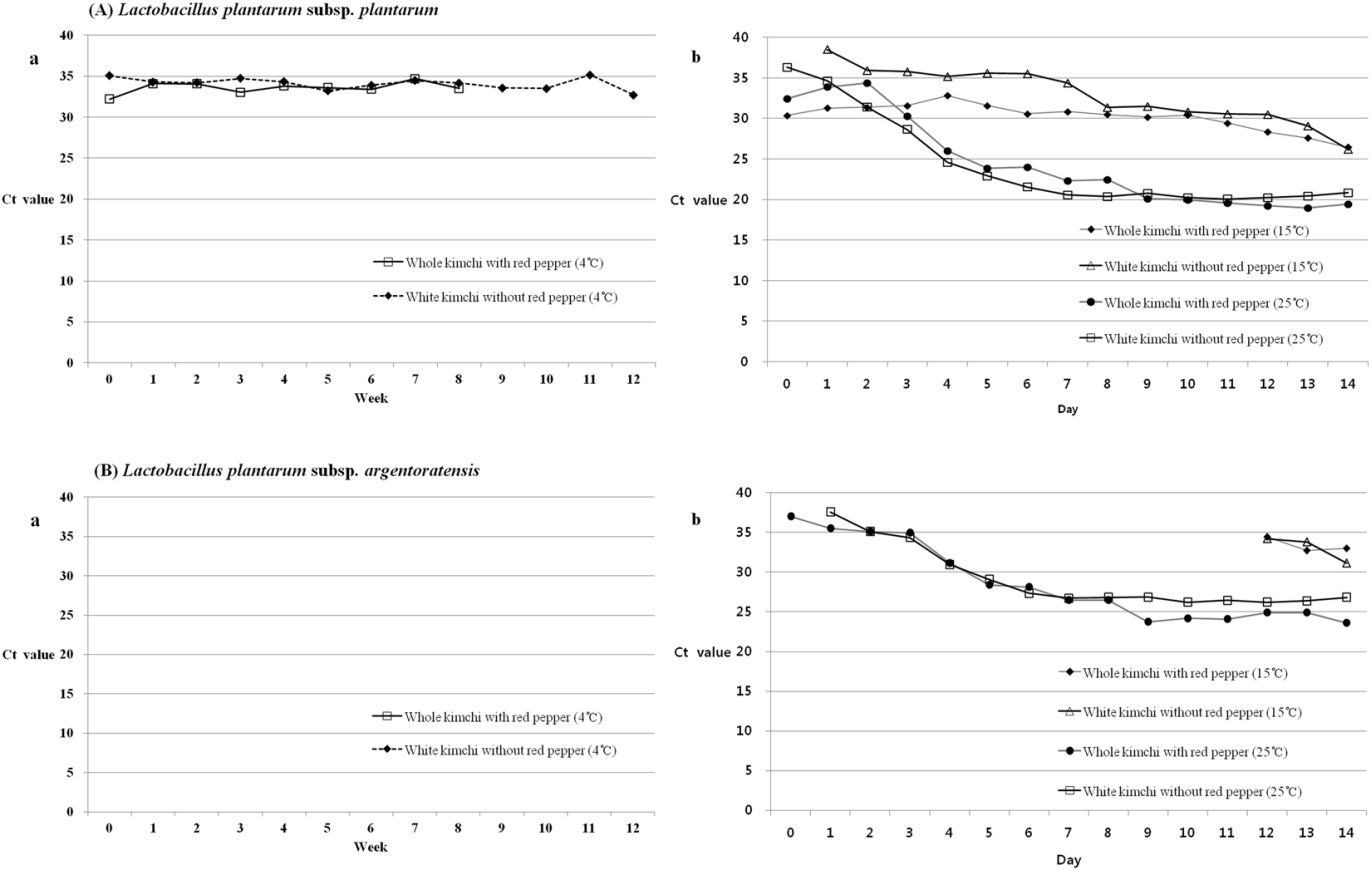
Changes in the real-time PCR Ct values during the quantification of *Lactobacillus plantarum* subsp. *plantarum* (**A**) and *Lactobacillus plantarum* subsp. *argentoratensis* (**B**) from total DNA isolated from salted Chinese cabbage kimchi fermented at 4 °C (a), 15 °C and 25 °C (b).

The difference in the proportion of *L. plantarum* subsp. *plantarum* was more apparent at 15 °C and 25 °C than at 4 °C during kimchi fermentation (Fig. 3A). All the kimchi samples stored at 4 °C presented the highest Ct values compared to the samples under other temperature conditions, but the corresponding kimchi samples at 15 °C or 25 °C exhibited the opposite results. Additionally, there was no significant change in density observed among the kimchi samples stored at 4 °C. Interestingly, starting from week 8, the occurrence of *L. plantarum* subsp. *plantarum* was not detected in whole kimchi (Chinese cabbage fermented with salt and red pepper powder) (Fig. 3Aa). Initially, the white kimchi (without red pepper powder) stored at 15 °C exhibited a greater delay in the fluorescence signal, which is indicative of the Ct value, than the whole kimchi samples under the same temperature conditions; however, from day 8, the density patterns of *L. plantarum* subsp. *plantarum* in both kimchi samples were almost the same.

In particular, all the kimchi samples stored at 25 °C exhibited the lowest Ct values and the most distinct changes in *L. plantarum* subsp. *plantarum* density (Fig. 3Ab).

For *L. plantarum* subsp. *argentoratensis*, unlike *L. plantarum* subsp. *plantarum*, all the kimchi samples stored at 4 °C showed no fluorescence curve for the Ct value (Fig. 3Ba). In addition, for the kimchi samples stored at 15 °C, the density of *L. plantarum* subsp. *argentoratensis* was much lower than that of *L. plantarum* subsp. *plantarum*, and this subspecies began to appear for the first time only after day 12 (Fig. 3Bb). For the samples stored at 25 °C, the density of *L. plantarum* subsp. *argentoratensis* was also much lower than that of *L. plantarum* subsp. *plantarum*, but the overall patterns of change in density were observed to be very similar.

## Discussion

*L. plantarum* is a typical *Lactobacillus* species that has not undergone adaptation to a particular environment. *L. plantarum* is a free-floating bacterium that acquires various functions that enable it to survive independently in particular hosts. In addition, recent clinical trials have shown that *L. plantarum* strains have the ability to reach the intestine and enhance the human immune system; this finding has received considerable attention because of the potential of this bacteria to be used as a vehicle for the delivery of biotherapeutics^17,18^. The probiotic properties associated with this species are mainly related to the promotion of human and animal health, and members of this species have been shown to reduce the levels of cholesterol and fibrinogen and the risk of cardiovascular disease and atherosclerosis^19^.

Currently, genomic approaches present innovative and noteworthy molecular methods aimed at studying microbial dynamics, for instance, glucose metabolism and interactions among microbial populations in a variety of environmental niches^1,20^.

Nonetheless, there is little high-quality evidence based on the use of species- or subspecies-specific molecular probes to precisely quantify the density of a targeted bacterium in different environments.

In general, DNA oligonucleotide probes, mostly targeting variable regions of the 16 S or 23 S rRNA genes, have been widely used for species identification and strain detection.

However, the most commonly used 16 S rDNA sequences are reported to unsuitable for all the species of lactobacilli because of the high identity (< 99%) shared by *L. plantarum* and *L. pentosus*^4^. Therefore, the oligonucleotide probe based on these DNA sequences cannot be used for *L. plantarum* identification since this probe cannot distinguish *L. plantarum* from *L. pentosus* and *L. paraplantarum*. Therefore, it is practically impossible to gain a comprehensive understanding of the changes in the abundance of a particular microbe with various types of food over time and how these changes affect the final quality of fermented products^15^. The critical pieces of information obtained regarding the ecological succession of these strains have been deemed necessary for the application of these strains in food biotechnology.

Recently, the ability to profile microbial communities via NGS has generated considerable interest for its use in the study of microbiomes. Groundbreaking studies have established experimental techniques and analytical frameworks for investigating microbiomes by sequencing a portion of a conserved sequence, for example, the 16 S rRNA gene, to quantify the microorganisms or operational taxonomic units (OTUs) that constitute a microbial community^10^. However, this system has significant drawbacks; such approaches are not appropriate for monitoring the ecological succession of a targeted bacterial species at the species level or lower. Therefore, molecular detection and quantification methods to analyse specific microbial communities are of great value.

In this study, we identified subspecies-specific genes using BLAST to analyse the population dynamics of *L. plantarum* subsp. *plantarum* and *L. plantarum* subsp. *argentoratensis*. The subspecies-specific primer sets were designed using the whole-genome sequences of *L. plantarum* subsp. *plantarum* ATCC 14917 (GenBank accession no. GCA_001434175.1) and *L. plantarum* subsp. *argentoratensis* DSM 16365 (GenBank accession no. GCA_001435215.1). The selected genes, including the gene encoding the cell surface protein for the subspecies-specific qPCR assay, were also confirmed to be highly variable among *L. plantarum* subspecies (Table 2).

Bacterial surface proteins constitute an amazing repertoire of molecules with important functions such as adherence, invasion, signalling and interaction with the host immune system or environment. In gram-positive bacteria, many surface proteins of the “LPxTG” family are anchored to peptidoglycans by an enzyme named sortase. Cell wall-anchored surface proteins, especially those with an N-terminal LPxTG-like motif, are reported to have involvement in adhesion in LAB as well as bacterial pathogens^21^. Adhesion to host cells is considered important for the persistence of *L. plantarum* as well as other LAB in the human gut and for the probiotic effects of these bacteria. Bacterial adhesion to the host mucosa is often mediated by the interaction of cell surface components, including receptor-specific binding and charge and hydrophobic interactions; mucosal and epithelial adhesion represent the early and late stages of adhesion, respectively^22^.

*L. plantarum* subsp. *plantarum* and *L. plantarum* subsp. *argentoratensis* are much more abundant at 15 °C or 25 °C than at 4 °C (Fig. 3). Consequently, temperature was confirmed to have a significant impact on the proportion of *L. plantarum*, whereas red pepper powder had little influence on the cell density of *L. plantarum* in kimchi, unlike *Weissella*^23^. At the subspecies level, the proportion of *L. plantarum* subsp. *plantarum* was much higher than that of *L. plantarum* subsp. *argentoratensis* regardless of fermentation temperature or the presence of red pepper powder. Interestingly, *L. plantarum* subsp. *argentoratensis* was not detected at 4 °C; it was detected only after day 12 at 15 °C.

Therefore, in contrast to previously reported data, *L. plantarum* was verified to be the dominant species and could ferment kimchi at temperatures as high as 15 °C^23,24^. In addition, *L. plantarum* was confirmed to not be a psychrophilic bacterium, being dominant at temperatures of 15 °C or higher. As shown in Fig. 3, the patterns for the changing populations of *L. plantarum* were very similar at 25 °C. The population of *L. plantarum* exhibited a slow increase until day 3 and then a sharp increase regardless of subspecies.

Consequently, we believe that this *de novo* assay may provide the possibility for subspecies-specific detection, identification and quantification of these *L. plantarum* strains in less time without the need for prior cultivation in various industries, including the fermented-food industry, where this method can be used to control ripening for quality-controlled food production, as it is crucial to precisely screen the changes in density of specific microbial communities during fermentation.

## Methods

### Bacterial strains and DNA isolation

A total of 36 LAB strains, including 24 lactobacilli strains, were obtained from the Korean Agricultural Culture Collection (KACC) and purchased from the Belgian Coordinated Collections of Microorganisms (BCCM). In total, 35 LAB strains were cultured at 30 to 37 °C for 48 hours on de Man, Rogosa, and Sharpe (MRS) agar (Difco, USA) and *Streptococcus gordonii* was anaerobically cultured on brain-heart infusion (BHI) medium (Difco, USA) solidified by the addition of 15 g/L Bacto-agar (BD, USA) at 37 °C in 90% N2/5% H2/5% CO2. All the strains were maintained in glycerol stocks at −80 °C. DNA was extracted from the corresponding strains listed in Table 2 as previously described and used to validate the specificity and sensitivity of the subspecies-specific oligonucleotide primers developed in this study^23,24^.

### Kimchi samples and DNA extraction

The two most common types of kimchi, white kimchi and whole kimchi, were purchased from a commercial factory in South Korea. Thirteen batches at 4 °C, 15 batches at 15 °C and 15 batches at 25 °C were stored for each type. At each sampling point, kimchi juice was sampled under aseptic conditions. The kimchi juice was centrifuged at 13,000 rpm for 10 min at 4 °C to pellet the bacteria. Total genomic DNA was extracted from the kimchi sample pellets using previously described methods^23–25^.

### Oligonucleotide primers and homology alignment for subspecies-specific PCR assay

The whole-genome sequences from *L. plantarum* subsp. *plantarum* str. ATCC 14917 (GenBank accession no. GCA_001434175.1), *L. plantarum* subsp. *argentoratensis* str. DSM 16365 (GenBank accession no. GCA_001435215.1) and closely related *Lactobacillus* strains were downloaded from the NCBI ftp site (ftp://ftp.ncbi.nlm.nih.gov/genomes/bacteria/) and compared in order to mine subspecies-specific genes for the quantitative detection of the two targeted *L. plantarum* subspecies using the method described by Chen and Lang with modifications^26,27^. The resulting candidate genes that presenting no substantial concordance with the other *Lactobacillus* strains were selected as quantitative PCR targets. The subspecies-specific primers for *L. plantarum* subsp. *plantarum* and *L. plantarum* subsp. *argentoratensis* were designed by Lasergene PrimerSelect software (version 7.2.1; DNASTAR Inc., USA) and synthesized by Bioneer Corporation (Daejeon, Korea) (Table 1). Each oligonucleotide primer set produced an expected amplicon from only the targeted subspecies. The nucleotide sequences of each primer set were evaluated for their specificity using the sequence alignment program BLAST^28^.

### Conventional PCR protocol

PCRs were performed in a PTC-225 thermocycler (MJ Research, Watertown, MA, USA). A reaction mixture with a final volume of 25 μl (1 × buffer, 0.2 mM each dNTP, 4.0 mM MgCl_2_) comprising 1.25 U of GoTaq^®^ Flexi DNA polymerase (Promega, Madison, WI, USA), 25 ng of template DNA and a 0.2 µM final concentration of each primer (Table 1) was prepared. The amplification reaction involved an initial denaturation at 95 °C for 5 min; 35 cycles of denaturation (95 °C for 1 min), annealing (30 s; *L. plantarum* subsp. *plantarum* at 65 °C, *L. plantarum* subsp. *argentoratensis* at 65 °C), and extension (72 °C for 1 min); and a final extension period of at 72 °C for 7 min. All the amplified products were resolved by 1.5% (w/v) agarose gel electrophoresis and stained with LoadingSTAR (DYNEBIO, Seoul, Korea). The gel was visualized using a VersaDoc 1000 gel imaging system (Bio-Rad Laboratories, USA).

### SYBR Green real-time PCR conditions

The SYBR Green qPCRs were performed in a CFX96 real-time PCR system (Bio-Rad Laboratories, USA). All qPCRs were carried out in a final reaction volume of 20 µl, containing 5 ng of purified DNA from each sample, the aforementioned primers (0.5 µM final concentration) and SYBR® Premix Ex Taq™ (TaKaRa Bio, Japan). The SYBR Green real-time PCR conditions were as follows: initial denaturation of 30 s at 95 °C; 40 cycles of 5 s at 95 °C, 30 s at 65 °C (30 s; *L. plantarum* subsp. *plantarum* at 65 °C, *L. plantarum* subsp. *argentoratensis* at 65 °C), and a melting curve analysis from 65 to 95 °C with an increment of 0.5 °C per 5 s.

Thermal cycling, data collection and data analysis were carried out with the Bio-Rad CFX Manager^TM^ version 1.6 software suite. The Ct value was defined as the PCR cycle at which the fluorescence signal exceed the background level.

A non-template negative control and standard amplification were included to confirm the quality of the SYBR Green real-time PCR assay. The LOQ and the limit of detection (LOD) were determined as the amounts of genomic DNA, plasmid DNA or bacterial cells in 10-fold serial dilutions of each subspecies (*L. plantarum* subsp. *plantarum* and *L. plantarum* subsp. *argentoratensis*) that corresponded to the threshold cycles at which the sum of the sensitivity and specificity of the assay was maximized. Standard curves enabled direct conversion of Ct values to gene copy number per microliter. The copy number of the template was calculated using the previously described formula^23,24,29^.

## Electronic supplementary material

Supplementary figure 1
