# Supplementary material for: New insight and metrics to understand the ontogeny and succession of Lactobacillus plantarum subsp. plantarum and Lactobacillus plantarum subsp. argentoratensis
Source: Sci Rep. 2018 Apr 16;8:6029. doi: 10.1038/s41598-018-24541-6 (PMC5902611; doi:10.1038/s41598-018-24541-6)

**Supplementary Information for**

**New insight and metrics to understand the ontogeny and succession of *Lactobacillus plantarum* subsp. *plantarum* and *Lactobacillus plantarum* subsp. *argentoratensis***

**Yong Ju Jin**^1^**, Yu Kyoung Park**^1^**, Min Seok Cho**^1^**, Eui Seok Lee**^2^ **& Dong Suk Park**^1^

^1^Department of Agricultural Biotechnology, National Institute of Agricultural Sciences, Rural Development Administration, Jeonju, 54874, Republic of Korea

^2^Department of Oral and Maxillofacial Surgery, Guro Hospital, Korea University, Seoul 08308, Republic of Korea

Correspondence and requests for materials should be addressed to Dong Suk Park (email: dspark@rda.go.kr).

**Supplementary figure 1.** **Specific PCR amplification of *Lactobacillus plantarum* subsp. *plantarum* and *L. plantarum* subsp.** ***argentoratensis* with the T1PL186F/R and LPA187 primer sets.** Agarose gel electrophoresis original photo.


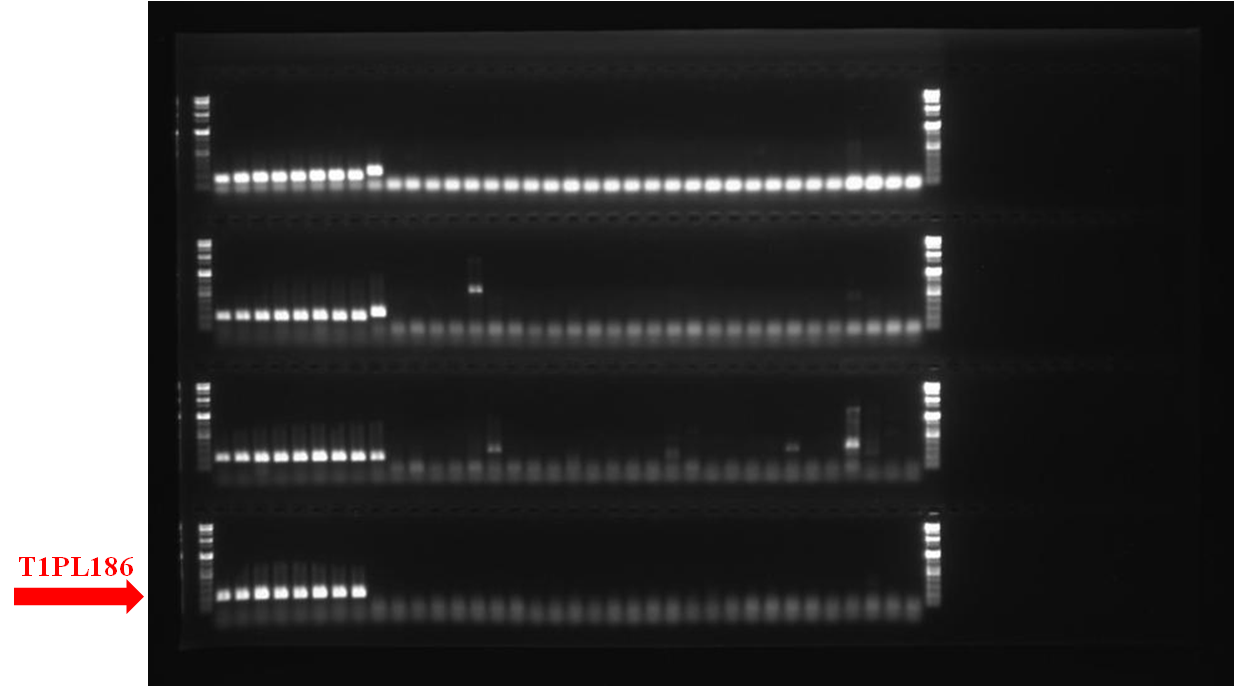

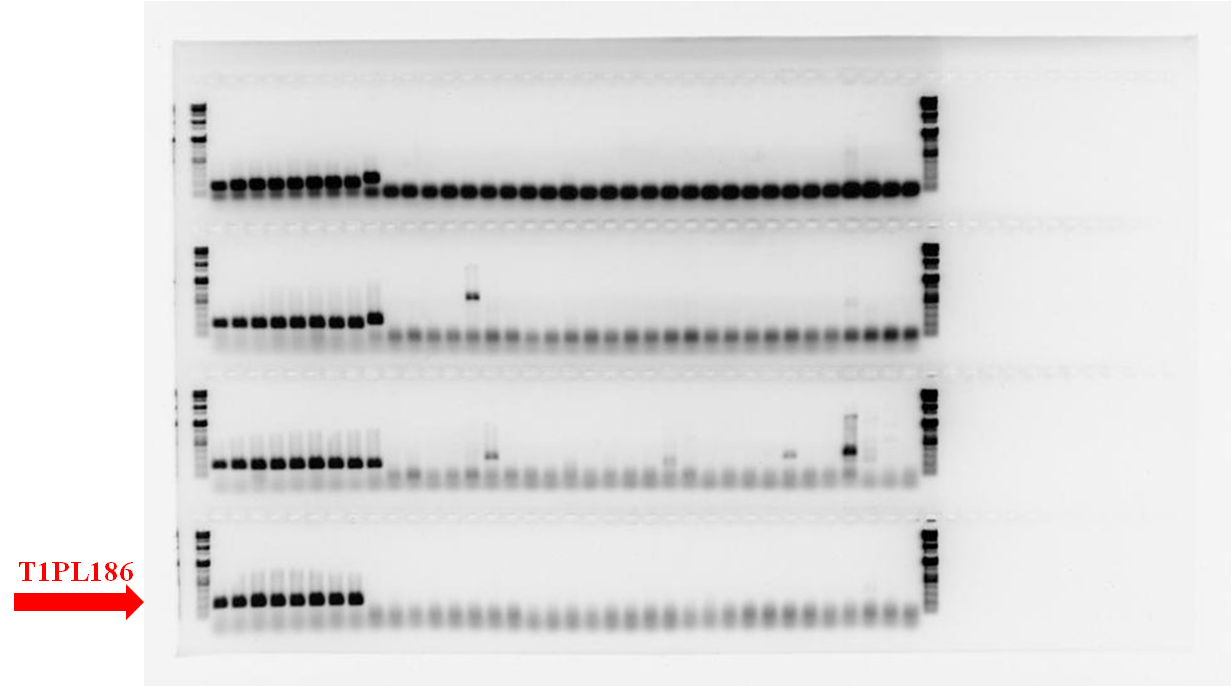

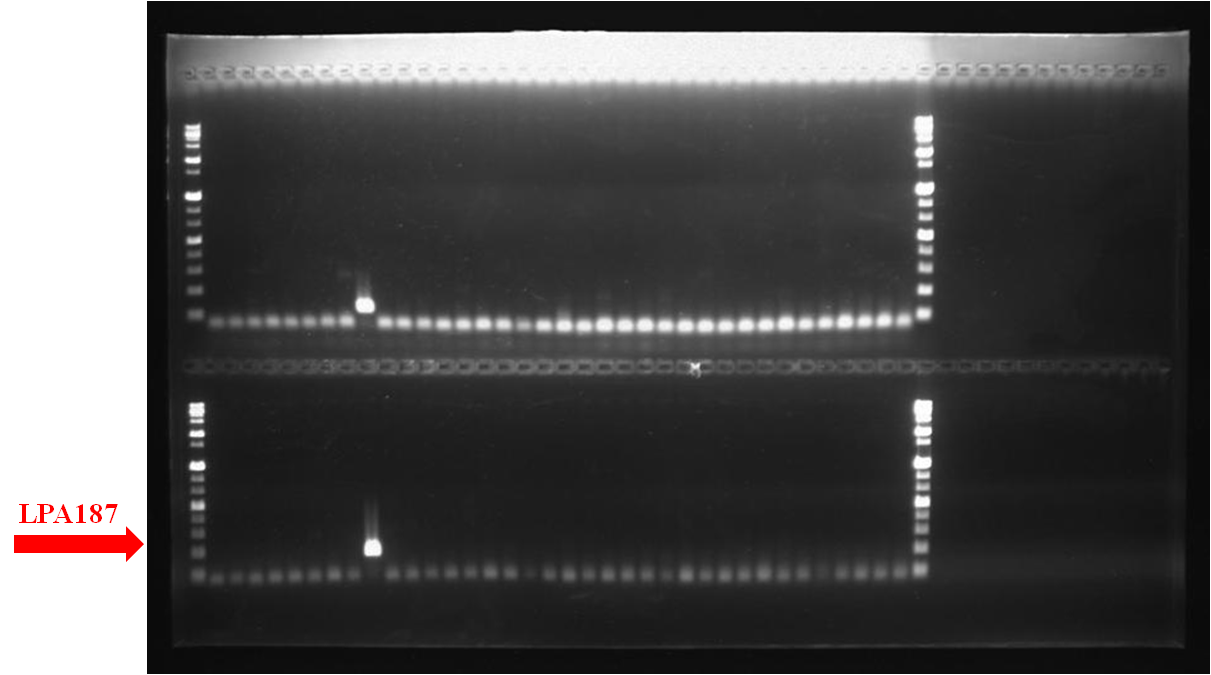

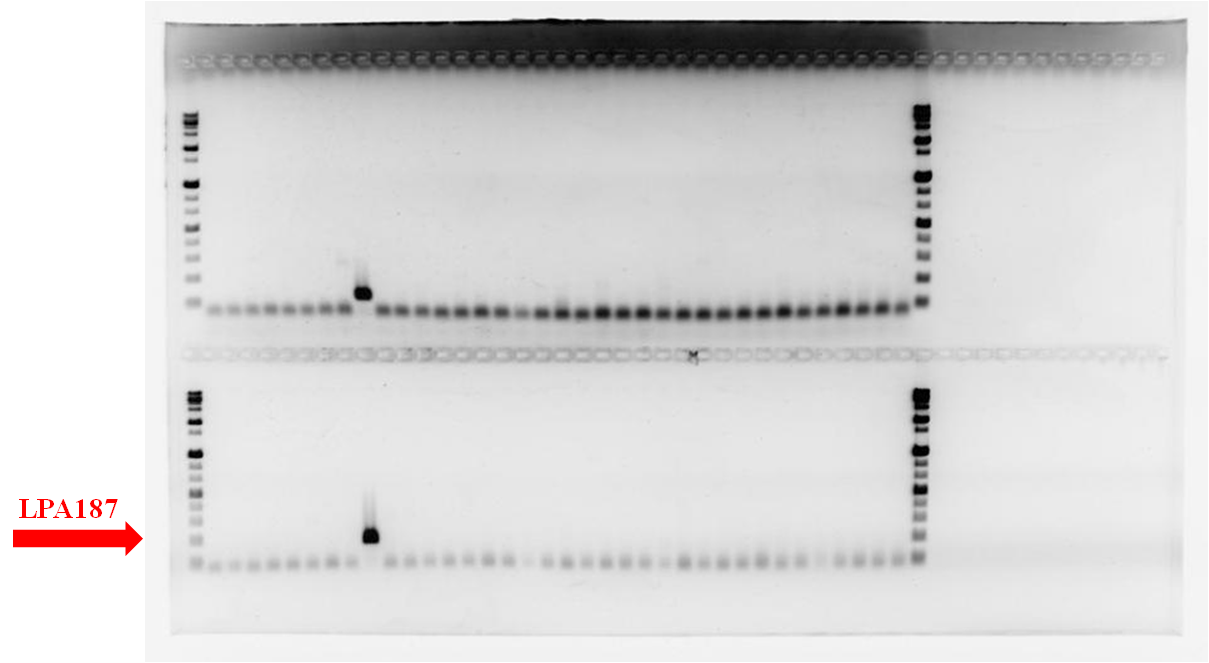

Supplement: Supplementary file 1 — Supplementary figure 1 [file 41598_2018_24541_MOESM1_ESM.docx]
